# Prevalence of Diarrhea, Feeding Practice, and Associated Factors among Children under Five Years in Bereh District, Oromia, Ethiopia

**DOI:** 10.1155/2022/4139648

**Published:** 2022-06-17

**Authors:** Yirgalem Feleke, Alemayehu Legesse, Meskerem Abebe

**Affiliations:** ^1^Kotebe Metropolitan University, Department of Public Health Nutrition, Addis Ababa, Ethiopia; ^2^Madda Walabu University, Department of Statistics, Bale Robe, Ethiopia

## Abstract

**Background:**

Diarrheal disease is a major public health problem among under-five children globally. In Ethiopia, it is the second cause of hospital admission and death among children under five years.

**Objectives:**

To assess the prevalence of diarrhea, feeding practice, and associated factors among children under five years in Bereh District, Oromia Special Zone Surrounding Finfine, Ethiopia.

**Methods:**

A community-based cross-sectional study was conducted among children less than 5 years old in Bereh District from May 15 to 29, 2021. A systematic random sampling technique was used to select a total of 455 study participants. Descriptive statistics were used to measure the prevalence of diarrhea as well as to summarize other study variables. A binary logistic regression model with an adjusted odds ratio and a 95% confidence interval (CI) was used to declare the associated factors with childhood diarrhea.

**Results:**

The prevalence of diarrhea was 17.3% in the past 15 days preceding the study period. About 53.4% of the mothers/caregivers were engaged in poor child feeding practices. Age of children [AOR = 9.146, 95% CI (2.055, 40.707)], birth order [AOR = 0.137, 95% CI (0.057, 0.329)], total family size [AOR: 5.042, 95% CI (2.326, 10.931)], not EBF [AOR: 4.723, 95% CI (1.166, 19.134)], prepare child foods separately [AOR: 0.252, 95% CI (0.091, 0.701)], feeding child immediately after cooking, handwashing method, and source of drinking water were significantly associated with under-five diarrhea.

**Conclusions:**

The prevalence of diarrhea among children under five is high. More than half of the participants were engaged in poor IYCF practice. Action targeting the factors associated with diarrhea should be taken to improve under-five child's health.

## 1. Background

According to the World Health Organization (WHO), diarrhea is defined as the passage of three or more loose or watery stools in a 24-hour period [1]. It is typically a symptom of an infection in the gastrointestinal tract caused by a variety of bacterial, viral, and parasitic organisms. It can last several days and deprive the body of the water and salts required for survival [2], resulting in severe dehydration and death or long-term consequences [3].

There are three main forms of childhood diarrhea, each of which can be fatal and necessitates a unique treatment regimen [4]. In an infected person, acute watery diarrhea is associated with significant fluid loss and rapid dehydration. Bloody diarrhea, also known as dysentery, is distinguished by visible blood in the stools. It is linked to intestinal damage and nutrient losses in infected people. Persistent diarrhea is an episode of diarrhea with or without blood that lasts at least 14 days [4].

Globally, diarrhea remains one of the major health problems [5]. In low- and middle-income countries (LMICs), diarrhea is still a leading cause of death and health loss among children under the age of five [6]. Evidence from Asian countries [7, 8] showed that early formula feeding increases the risk of childhood diarrhea. Similar evidence from sub-Saharan African countries [9] has indicated that early introduction of complementary foods and bottle-feeding was associated with the onset of diarrhea among infants and young children. This could be due to the substitution of complementary foods for irreplaceable human milk, as well as contamination of the food and/or the bottle's nipple [10].

In Ethiopia, diarrhea is a major public health problem and the second leading cause of clinical presentation at health facilities among children under the age of five, after pneumonia, and the prevalence is higher in rural areas than in urban areas due to a variety of factors, including feeding practices [11]. However, evidence is scarce regarding under-five children's diarrheal disease and feeding practices in Bereh District.

Evidence in LMICs [9, 12] has shown that early initiation of breastfeeding (EIBF) and exclusive breastfeeding (EBF) was protective against diarrhea. It is because breast milk contains all anti-infective and essential nutrients necessary for children's growth and development [13].

On the other hand, early discontinuation of EBF and early introduction of complementary feeding were linked to childhood diarrhea [14]. However, evidence suggests that infants and young children who were primarily breastfed were less likely to experience diarrhea [15]. In Ethiopia, sociodemographic factors, hand hygiene practices, breastfeeding status, and the timing of complementary food initiation are some of the causes of childhood diarrhea [11]. Similarly, local evidence [16–23] revealed that failure to practice appropriate infant and young child feeding is significantly associated with childhood diarrhea.

However, there is a lack of scientific evidence on the prevalence of diarrhea, feeding practice, and associated factors in the study area. Therefore, this was study aimed at assessing the prevalence of diarrhea, feeding practice, and associated factors.

### 1.1. Justification of the Study

Despite significant reductions in childhood mortality and morbidity in Ethiopia, many children die from diarrhea before their fifth birthday [24]. This is because factors such as accessible, safe, and adequate water supply; environmental sanitation and personal hygiene; the availability and quality of MCH services and facilities; maternal education level; feeding practice; and place of residence can all have a significant impact on diarrhea morbidity and mortality.

However, (i) in Bereh District, there is no scientific evidence related to childhood diarrhea and feeding practice, but it is considered a special zone. (ii) According to a master's student of Addis Ababa University, evidence (unpublished) showed that the rural water supply coverage of the District is 34.6% and sanitation coverage is 14.8% [25]. (iii) There is a rebounding of childhood diarrhea prevalence in rural areas of Ethiopia [21, 22, 26], when compared to EDHS 2016 [11]. This suggests that many more studies are remaining to be done on child saving from diarrheal morbidity and mortality.

Thus, the purpose of this study was to determine the prevalence of diarrhea, feeding practices, and associated factors among children under the age of five at the community level in Bereh District, Oromia Special Zone Surrounding Finfine.

## 2. Methods and Materials

### 2.1. Study Area and Period

The study was conducted in Bereh District from May 15 to May 29, 2021. Bereh is one of Ethiopia's districts in the Oromia Region. It was a part of the former Berehna Aleltu woreda, which was divided into Aleltu and Bereh District, as well as Sendafa Town. Bereh is located in the Oromia Special Zone Surrounding Finfine and is bounded to the south by the Akaki and east Shewa Zones, to the southwest by Addis Abeba, to the west by Sululta, to the north by the north Shewa Zone, and to the east by the Amhara Region.

This district's landscape has been described as undulating mountains covered by a scattered settlement pattern, making development efforts more difficult. Agriculture is the district's primary source of income. A household's average land size ranges from 1 to 2 hectors, and the major crops grown in the area include wheat, teff, barley, field peas, lentil, chickpea, vetch, and oilseeds. The productivity of wheat, barley, teff, beans, and pea is 7, 6, 6, 4, and 5 quintals/hector, respectively. The national average for cereals (11.54 quintals/hector) and pulses (6.74 quintals/hector) is 5.24 and 2.24 quintals/hector, respectively, higher than the district productivity [27]. The district's rural water supply and sanitation coverage rates are 34.6 percent and 14.8 percent, respectively [25]. This easily demonstrates that the socioeconomic status of this area is poor.

According to the 2007 national census, this district had a total population of 80,808, of which 41,023 were men and 39,785 were women; none of its residents lived in cities. And the population is growing at a rate of 2.9 percent [28]. According to the district health office annual report and statistical data 2020 (unpublished), the total population is 99,669, of which 50,831 are women, 48,838 are men, and 14,452 are children under the age of five. It had 24 primary schools and only one secondary school in Sendafa town. The district is divided into 22 rural kebeles. There are four governmental health centers and 20 health posts in the district. Health extension workers provide health care at the kebele level [29].

### 2.2. Study Design

A community-based cross-sectional research design was used.

### 2.3. Population

#### 2.3.1. Source Population

The source of the population was all mothers/caregivers with children under the age of five in the Bereh District.

### 2.4. Eligibility Criteria

#### 2.4.1. Inclusion Criteria

All mothers+/caregivers of children under five years who lives in Bereh District were included in the study.

#### 2.4.2. Exclusion Criteria

A mother/caregiver who is critically ill and a stranger to the district was excluded from the study.

### 2.5. Sample Size and Sampling Procedure

**Figure 1:**
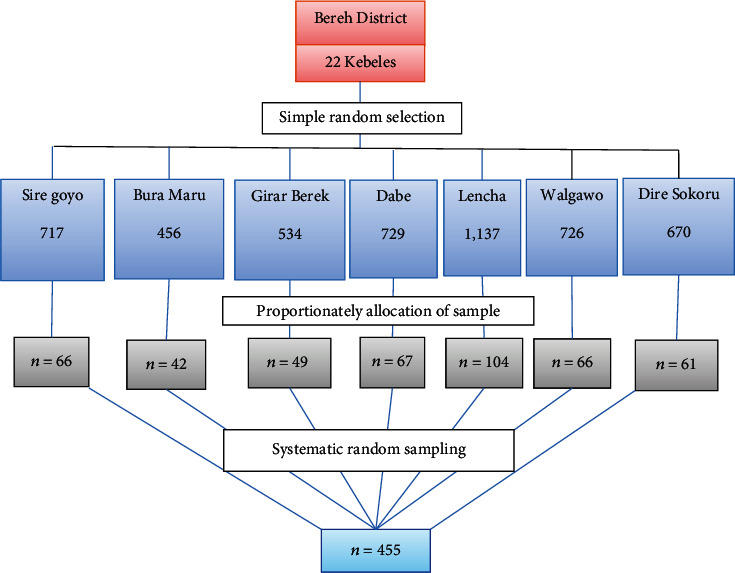
Sampling procedures for the prevalence of diarrhea, feeding practice, and associated factors among children under five years in Bereh District, Oromia Special Zone Surrounding Finfine, Ethiopia, May 15-29/2021 (*n* = 455).

Using a single population proportion, the sample size (455) was calculated. The district's kebeles and households with children under the age of five were chosen using a two-stage sampling method. First from 22 kebeles in the district, 7 kebeles were selected by using simple random sampling methods. After allocating a proportionate sample size to each selected kebele, the mother/caregiver pairs were chosen using a systematic random sampling technique. One child-mother/caregiver pair was selected by simple random sampling method by starting point from near the health post for each kebeles to collect data about diarrhea and feeding practice using pretested structured interview questionnaire. In the case of households with two or more children under five years, one child was selected by the lottery method. Following the selection of the first household at random, subsequent households were chosen at 10th household intervals. When the selected households have no children under five years, the next neighborhood household was selected.  Figure 1 is briefly illustrating the sampling procedure of the study.

### 2.6. Variables Included in the Study

Prevalence of diarrhea is considered as an outcome variable, whereas sociodemographic (age, sex of the child, birth order, education level, occupation, family size, source of drinking water, and income), breastfeeding (prelacteal feeding, early initiation of breastfeeding, exclusive breastfeeding, predominant breastfeeding, and continued breastfeeding at two years), introduction of complementary feeding (time of introduction to complementary foods, type of foods, feeding uncooked food, immediately feeding cooked food, hand hygiene habit, and feeding material washing habit), and feeding method (spoon, bottle, and hand) are explanatory variables.

#### 2.6.1. Operational Definitions

Diarrhea: the proportion of children under the age of five who had three or more loose or watery stools every twenty-four hours during the previous two weeks of data collection, as reported by the children's mothers/caregivers. For this study, diarrhea is classified as yes and was coded as 1 (for those children who experienced diarrhea), and no was coded as 0 (for those children who did not experience diarrhea) (for those children who has no experienced diarrhea in the past two weeks period of the data collection)

Good feeding practice: defined as a score of ≥75% out of 8 questions and poor feeding practice if scored <75% out of 8 questions [30, 31]

Early or timely initiation of breastfeeding: the proportion of children under five years who were put to the breast within 1 hour of birth

Exclusive breastfeeding: the proportion of children who receive only breast milk for the first six months of their lives and no other solid or liquid food, except vitamins, minerals, and medicines

Prelacteal feeding: the proportion of children who receive any foods (such as water, honey, butter, tea, formula milk, and fruit juice) given before the onset of lactogenesis II, that is, the onset of copious breast milk secretion occurring within 3 days of birth

Bottle-feeding: the proportion of children under five years who received any liquid (including breast milk) or semisolid food from a bottle with a nipple

Handwashing during critical time: measured as yes, refers to the proportion of caregivers' handwashing practice after utilization of latrine, before food preparation, and child feeding as identified by caregivers' oral report of their practice. If at least one is missed from a critical time, it is considered as no

Source of drinking water: categorized as improved if it is from stand pipe, protected spring, and tap water while unimproved if it is from the river, pond, or unprotected spring

### 2.7. Data Collection Method

Standardized questionnaires were adopted from WHO and EDHS [11, 31] and were first modified in English, then translated into local languages (Afan Oromo and Amharic), and finally translated back into English by another person fluent in both languages to ensure consistency and capture relevant information. Seven trained diploma holder nurse data collectors administered the questionnaire. To obtain reliable information, face-to-face interviews were conducted with nurses as interviewers to facilitate communication between interviewers and respondents. However, for the study participant who refused to answer any specific questions at any time during the interview, it was recorded as missing and the questionnaire was recorded as incomplete.

### 2.8. Data Quality Management

To ensure the quality of the data, the principal investigator first trained the data collectors and supervisors for one day on the objectives, relevance of the study, interviewing methods, the confidentiality of information, and informed consent. Before beginning the data collection process, data collectors had to explain the purpose of the research (to better understand and indicate the occurrence of diarrhea and feeding practice) and obtain an honest response from respondents before moving on to the research questions. To ensure consistency, the data collection tool was modified in English, translated into Afan Oromo and Amharic, and then returned to English. The questionnaires were pretested in Lega Beri Kebele, on 5% of the sample size (24 participants) of mothers with children under the age of five living in Bereh District. To ensure accuracy, a pretest was performed before the actual data collection work to check how respondents understood the questionnaire and to estimate the time needed. Then, a questionnaire was adjusted and modified accordingly based on the findings of the pretest.

Before entering data, each questionnaire was checked for completeness. To reduce errors, the principal investigator coded each completed questionnaire on a prearranged coding sheet. The supervisors in the field then checked them every day and rechecked them for consistency, clarity, and completeness on the same day. The overall activities of the study project are closely observed and coordinated by the principal investigator. Supervision was carried out during data collection to understand how the data collectors handled the questionnaire, and each completed questionnaire was checked daily for completeness, accuracy, clarity, and consistency. If there were any gaps, corrective measures were taken, special care was taken during data entry and cleaning, and the entire data set was cross-checked for reliability before analysis. Finally, the principal investigator analyzed it with the assistance of an experienced expert statistician.

### 2.9. Data Processing and Analysis

The principal investigator checks the data before entering it at the end of data collection. After cleaning, coding instructions and data entry were completed using EPI-Data version 3.1, and data cleaning and analysis were completed using SPSS version 21 software. Descriptive statistics were used to determine the prevalence of diarrhea as well as to summarize other study variables. A binary logistic regression model was used to examine the relationship between childhood diarrhea and explanatory variables. A variable with a *p* value less than 0.25 in the bivariate analysis was moved to multivariable analysis. VIF with a cut-off point of 10 was used to check for multicollinearity error. Furthermore, the Hosmer-Lemeshow goodness fit was used to assess model adequacy with a cut-off point *p* value greater than 0.05. In this study, the model fits the data well because the *p* value of the Hosmer-Lemeshow goodness fit was 0.657, and the model well fits the data. In the final model, a *p* value of less than 0.05 and an adjusted odds ratio with a 95% confidence interval (CI) were used to declare the associated factors.

## 3. Results

### 3.1. Sociodemographic and Health Service Characteristics of the Respondents

The study included 455 mother/caregiver-child pairs, with a complete response rate of 440 (96.7 percent) from all respondents. The respondents' mean age was 29.89 (5.855 SD), ranging from 15 to 50 years old, with the majority of them 280 (63.6 percent) being between the ages of 25 and 34. The majority of the respondents 384(87.3 percent) were Oromo by ethnicity, and approximately 375 (85.2 percent) were orthodox by religion. The majority of the mothers/caregivers (218/49.5%) had completed primary school. The majority of the participants 408 (92.7%) were married, with 381 (86.6%) working as housewives as their primary occupation. Four hundred thirteen mothers (93.9 percent) received ANC, and 283 (64.3 percent) gave birth at a health facility. About 353 (80.2 percent) of the respondents had one or more children under the age of five, and the average family size of the households was 4.94 (1.247 SD) people, with the majority of them 336 (76.4 percent) having less than or equal to five members. In terms of husband's educational level, 259 (58.9 percent) were primary school graduates and 330 (75.0%) were farmers by occupation. Most of the 401 (91.1%) lived together with their family. More than half of the respondents 253(57.5%) earned less than one thousand birrs per month (Table 1).

### 3.2. Sociodemographic and Health Service Characteristics of Children

Approximately half of the children 221(50.2 percent) were females, with the majority of the children 172 (39.1 percent) being between the ages of 24 and 35 months. The majority of the children (345, or 78.4 percent) were two or above by birth order, and 419, or 95.2 percent, were vaccinated against Rota virus (Table 2).

### 3.3. Prevalence of Diarrhea among Children under Five Years

In this study, 17.3 percent of the children (95 percent CI: 13.9–21.1) had diarrhea in the 15 days before the study period (Table 3).

### 3.4. Feeding Practice

**Figure 2:**
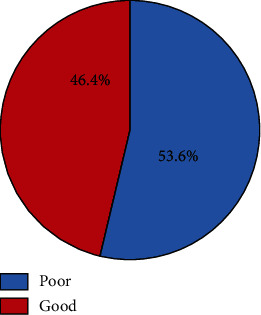
Feeding practice among children under five years in Bereh District, Oromia, Ethiopia, 2021.

This study indicates that more than half of the participants (236 (53.6%)) practice poor feeding of their children (Figure 2).

### 3.5. Breastfeeding Practice

Among all participants, 434 (98.6%) breastfed their children, with 209 (47.5%) exclusively breastfeeding. Breastfeeding was delayed for more than half of the 228 respondents (51.8 percent). Prelacteal feeding was given to 41 participants (9.3%) for their children, with cow's milk accounting for 20 (4.5%) of the prelacteal feeding ingredients, while the remaining 12 (2.7%), 3 (0.7%), and 6 (1.4%) were butter, honey, and others. In terms of breastfeeding duration, 186 (42.3 percent) of the participants breastfed their children for two years or more (Tables 4 and 5).

### 3.6. Complementary Feeding

Almost half of the 222 participants (50.5 percent) began complementary feeding for their children when they were less than 6 months old. The majority of the participants (331 (75.2 percent) prepared their children's food separately, and 182 (41.4 percent) fed porridge-type food. Approximately half of the participants, 219 (49.8%), were feeding their children with a cup/spoon (Table 6).

#### 3.6.1. Hygienic Related Child Feeding Practice

The majority of the participants (372(84.5 percent) did not feed their children unwashed fruits, while the remaining 68 (15.5 percent) did. Three hundred sixty-eight respondents (83.6 percent) did not feed their children uncooked foods, while 72 (16.4 percent) did. The majority of the mothers or caregivers (308 or 70.0 percent) washed their hands during a critical time. Three hundred five respondents (69.3 percent) used soap and water for handwashing, while the remaining 135 (30.7 percent) used only water. In terms of drinking water, the majority of the respondents 270 (61.4 percent) obtained it from a better source. Only 61 (13.9 percent) of the participants treated their drinking water at home (Tables 4 and 7).

### 3.7. Factors Associated with Childhood Diarrhea

#### 3.7.1. Univariate Analysis

In the bivariate analysis at a 25% level of significance, the number of under-five children in the house, age of children, birth order, children immunized for Rota virus, mothers' educational level, total family size, child feeding practice, breastfeeding, history of BF in the first 6 month of life, breastfeeding initiation time, duration of EBF and period of BF continuation, age at complementary feeding, prepare child food separately, and feeding method, feeding uncooked food, feeding cooked food immediately, handwashing at a critical time, handwashing method, and source of drinking water were identified as statistically significant with childhood diarrhea. However, the rests have no association with the occurrence of diarrhea at this level of significance.

#### 3.7.2. Multiple Covariate Analysis

Multiple covariate binary logistic regression analysis revealed that children from families with two or more under-five children in the house were 2.2 times more likely to develop diarrhea [AOR: 2.204, 95 percent CI (1.082, 4.488)] than children from families with one under-five child in the house. The odds of developing diarrhea were 9 times higher in children aged 7-11 months [AOR: 9.146, 95 percent CI (2.055, 40.707)] than in children aged 48 months. Similarly, children aged 12-23 months were 4.9 times more likely to experience diarrhea [AOR: 4.979, 95 percent CI (1.206, 20.56)] than children aged 48 months. Being the second and above by birth order was 7.3 times less likely to develop diarrhea [AOR: 0.137, 95% CI (0.057, 0.329)] as compared to the first birth order. Children from more than five family sizes were 5 times more likely to suffer from diarrhea [AOR: 5.042, 95% CI (2.326, 10.931)] when compared to children from five or fewer family sizes.

According to this study, children who had not EBF during their first 6 months of life were 4.7 times more likely to experience diarrhea [AOR: 4.723, 95% CI (1.166, 19.134)] as compared to children who had exclusive breastfeeding during their first 6 months of life.

Children from mothers/caregivers who had to prepare child foods separately were 3.9 times less likely prone to diarrhea [AOR: 0.252, 95% CI (0.091, 0.701)] when compared to their counterparts.

Children who ate food after cooking immediately were 2.8 times less likely to be affected by the diarrheal disease [AOR: 0.361, 95% CI (0.171, 0.762)] as compared to children who had not eaten foods after cooking immediately. The risk of childhood diarrhea was higher among children whose mothers/caregivers washed their hands with water only during the critical time [AOR: 2.949, 95% CI (1.341, 6.482)] when compared with mothers/caregivers who had to wash their hands with water and soap. Children from a family who had to get drinking water from the improved source were less likely prone to diarrhea [AOR: 0.472, 95% CI (0.235, 0.949)] as compared to children from a family who had to get drinking water from the unimproved source.

In multivariate analysis, mother's educational level, breastfeeding, breastfeeding initiation time, duration of EBF, period of BF continuation, age at CF, feeding method, handwashing at critical time, and feeding practice did not show any statistically significant association with under-five children diarrhea (Table 8).

## 4. Discussion

The purpose of this study was to determine the prevalence of diarrhea, feeding practices, and associated factors among children under the age of five in Bereh District, Oromia, Ethiopia. The prevalence of diarrhea in this study was 17.3 percent (95 percent confidence interval: 13.9–21.1 percent), which is comparable to studies done in Nigeria 21.1 percent [32], Farta Woreda, North West Ethiopia 16.7 percent [33], Kamashi District, Western Ethiopia 14.7 percent [23], Bahir Dar city, Northwest Ethiopia 14.5 percent [34], and Debre Berhan town 16.4 percent [34]. However, the results of this study were lower than those of the previous studies in Sana'a, Yemen (29.07 percent), Cameroon, sub-Saharan Africa (26.1 percent), Mbour, Senegal (26.1 percent), Nyarugenge District, Rwanda (26.7 percent), Pader District, Northern Uganda (29.1 percent), Benna Tsemay District, Southern Ethiopia (23.5 percent), Rural Areas of North Gondar, Ethiopia (22.1 percent), and Harena Buluk [26]. The current finding is also relatively high when compared to an Indian study. The current finding also relatively high compared to a study done in India 9 percent [35] and Dale District, Southern Ethiopia 13.6% [19]. The variation might be due to the study period or differences in the study year, family sizes, and infant and young child feeding practices. It indicates that still many children are suffering from easily preventable diarrheal disease and so much work is left to prevent or decrease childhood diarrhea.

In this study, 53.4 percent of the mothers/caregivers practiced poor child feeding. The results of this study are similar to those in the Benenzamay district of Ethiopia (54.3% of mothers/caregivers are said to have poor feeding habits) [36]. It demonstrates that poor IYCF practice continues to affect more than half of the children. One possible explanation is that mothers and caregivers are not aware of appropriate IYCF practices.

According to the findings of this study, 47.5 percent of participants exclusively breastfed their children during the first six months of life. The findings were consistent with a study conducted in Tanzania (48.6 percent) and Bahir Dar city administration in Ethiopia (49.1 percent) [37]. This study's findings are relatively higher than those of previous studies in the Sidama Zone, Southern Ethiopia (13.2 percent), and Addis Ababa (44.2 percent) [38]. However, the findings of this study are lower than those of the previous studies in Ethiopia (60.1 percent) and Guji Zone, Oromia Regional State, Ethiopia (58.9 percent) [17]. Furthermore, the current result is significantly lower than the Ethiopian government's five-year health service transformation plan, which was aimed at achieving 72 percent exclusive breastfeeding (EBF) nationwide by 2020 [39]. The difference could be attributed to mothers' educational level and lack of knowledge about exclusive breastfeeding. Health promotion and education as well as having a written infant feeding policy that is routinely communicated to health worker staff and parents might reverse the problems.

Less than half of the mothers, 48.2 percent, practiced early breastfeeding initiations at birth. This result was higher than the 43.4 percent found in the Tanzanian study [40]. Furthermore, the current finding was lower than that of the Ethiopian study, which found 66.5 percent [41], and in Addis Ababa, Ethiopia, which found 52.6 percent [38]. This lower finding could be attributed to mothers' lack of knowledge about the benefits of colostrum from breast milk, which is high in minerals and vitamins.

For infants and young children aged 6 to 24 months, optimal complementary feeding with continued breastfeeding is critical to ensure they are healthy, well-nourished, and better able to survive an episode of diarrhea [3]. However, according to the findings of this study, approximately half of the participants (50.5 percent) began complementary feeding for their children when they were less than 6 months old. This result was lower than the 53.3 percent found in a study conducted in Southwestern Saudi Arabia [42]. This finding, however, was higher than that of a study conducted in Southern Nepal (34.5 percent) [43], Northwest Ethiopia (4.9 percent) [21], and West Guji Zone, Oromia Region, Ethiopia (21.8 percent) [17]. This could be due to mothers' lack of knowledge about proper feeding practice and timing. Having written infant and child feeding policies that are routinely communicated to all health workers, social worker affairs staff, educational worker staff, and parents may be a solution to these issues.

The current study discovered that the number of children under the age of five in the household was statistically associated with childhood diarrheal disease. It implies that children from families with a large number of under-five children in the house have a higher risk of developing diarrhea than children from families with only one under-five child in the house. This study is consistent with the previous research conducted in Harena Buluk District, Oromia Region, South East Ethiopia [26], Medebay Zana District, Northwest Tigray, Ethiopia [44], and Benishangul Gumuz Regional State, North West Ethiopia [45, 46] This could be due to the mother's or caregiver's inability to care for a large number of children. As a result, effective child birth spacing would play a significant role in the prevention of childhood diarrhea.

Children aged 7 to 11 months were at a higher risk of developing diarrhea than children aged 48 months. Likewise, when compared to children 48 months of age, children aged 12 to 23 months were more likely to develop diarrhea. This finding was consistent with the findings of studies in Southwestern Saudi Arabia [42], Sana'a, Yemen [47], rural Ethiopia [2], Debre Berhan town, Ethiopia [16], and Benishangul Gumuz, North West Ethiopia [46]. This could be because maternal antibodies are declining or children's immunity is not mature enough to protect against infection, as well as the introduction of complementary foods that could be contaminated by various pathogenic organisms. Also, at this age, children begin crawling and testing everything by mouth, making them susceptible to pathogenic in the environment that causes diarrhea. It implies that because children of these age groups are more vulnerable, they need special care.

The current study found that birth order affected the occurrence of diarrhea in children under the age of five. When compared to the first birth order, those born in the second and higher birth orders were 7.3 times less likely to develop diarrhea. The current finding is comparable to studies conducted in Southwestern Saudi Arabia [42], rural Ethiopia [2], and Benishangul Gumuz Regional State, as well as northwest Ethiopia [46]. This could be due to the mothers'/caregivers' lack of experience and quality of care during the first childbirth. As a result, it is possible to speculate that educating first-time pregnant mothers on how to provide special care to their baby after birth may have a positive impact on diarrhea prevention.

This study's findings also revealed that family size was significantly associated with diarrhea among children under the age of five, a finding that was supported by studies conducted in Sana'a, Yemen [47] and Pader District, Northern Uganda [48]. This could be because overcrowding makes caregiving more difficult and facilitates cross-contamination. Family planning could have been a viable solution to this problem.

Breastfeeding exclusively is universally recommended as the best way to feed infants during the first six months of life because it provides critical nutrients, antioxidants, hormones, and antibodies required by a child to survive and develop [3]. Infants who are exclusively breastfed for the first six months of life and continue to be breastfed until the age of two and beyond have fewer infections and less severe illnesses than those who are not [4]. Children who were not exclusively breastfed in their first six months were more vulnerable to diarrhea in this study, which is consistent with other studies conducted in Indonesia [49], India [50], Rural Vietnam [51], Southwestern Saudi Arabia [42], Tanzanian [40], Kaduna north local government area, Nigeria [32], Ethiopia [15], Medebay Zana District, Ethiopia [44], West Guji Zone, Oromia Region, Ethiopia [17], and Somali Region, Eastern Ethiopia [17, 52]. It shows that many children are still suffering from diarrhea.

This study also found that hygienic child feeding practices were statistically associated with childhood diarrheal disease. Diarrhea was less likely to occur in children whose mothers/caregiver prepared separate meals for the child and fed the child immediately after cooking. This finding is consistent with the research conducted in Pader District, Northern Uganda [48], Southern Nepal [43], and Hadaleala District, Afar Region, Northeast Ethiopia [53]. Stored food after cooking may increase the risk of contamination with a variety of pathogens that cause diarrheal disease. As a result, preparing child foods separately and feeding immediately after cooking may reduce the possibility of complementary food contamination and act as a protective measure against diarrhea.

This study also discovered that diarrhea was very common among children of mothers/caretakers who washed their hands only with water at critical times. This finding is consistent with findings the from studies in Kamashi District, Western Ethiopia [23] and Harar Town, Eastern Ethiopia [18], which found that poor handwashing and method of handwashing (only with water) before preparing and feeding the child were significantly associated with diarrhea. This implies that mothers/caretakers should always wash their hands with water and soap at critical times to avoid the occurrence of childhood diarrhea.

The current study found that children whose mothers drank water from an unimproved source experienced more diarrhea than children whose mothers drank water from an improved source. This study's findings are consistent with those of many other studies conducted in Pader District, Northern Uganda [48], Jamma District, South Wello Zone, Northeast Ethiopia [54], and Medebay Zana District, Northwest Tigray, Ethiopia [44]. This may because the water source is easily contaminated with environmental wastes, particularly human feces. It suggests that a health promotion intervention should target mothers or caregivers who get their drinking water from unimproved sources to introduce a home-based drinking water treatment system and reduce childhood diarrheal morbidity.

## 5. Conclusion

According to the findings of this study, the prevalence of diarrhea among children under the age of five was 17.3 percent, which is considered high. More than half of the participants are practicing poor IYCF. The proportion of children exclusively breastfed and early initiation of breastfed were much lower compared to the HSTP plan. A large proportion of children begin complementary feeding before the age of six months. The number of children under the age of five, the age of the children, the size of the family, the birth order, breastfeeding status in the first 6 months, preparing child food separately, feeding food immediately after cooking, handwashing method, and source of drinking water were all found to be significantly associated with childhood diarrhea. Finally, the district health office should incorporate and prioritize health education and counseling to increase targeted mothers' awareness and knowledge of the importance of family planning, infant and young child feeding practices, handwashing methods during a critical times, and home-based drinking water treatment in its annual planning and follow-up accordingly through receiving reports and considering those significant factors.

### 5.1. Limitation of the Study

Since the study was a community-based cross-sectional study design, the reporting was not confirmatory rather it was the oral report of mothers/caregivers.

## Figures and Tables

**Table 1:** Sociodemographic and health service characteristics of mother/caregivers in Bereh District, Oromia, Ethiopia, 2021.

Variable	Category	Frequency (*n* = 440)	Percentage (%)
Number of under-five children in the house	One	353	80.2
	Two and above	87	19.8
Total family size	≤5	336	76.4
	>5	104	23.6
Mother/caregiver age	≤24 years	73	16.6
	25-34 years	280	63.6
	≥35 years	87	19.8
Mother/caregiver educational level	Not educated	105	23.9
	Primary	218	49.5
	Secondary and above	117	26.6
Ethnicity	Oromo	384	87.3
	Amhara	36	8.2
	Others	20	4.5
Religion	Orthodox	375	85.2
	Muslim	16	3.6
	Protestant	9	2.0
	Others	40	9.1
ANC follow-up	No	27	6.1
	Yes	413	93.9
Place of delivery	Home	147	33.4
	Health institution	283	64.3
	Others	10	2.3
Mother's occupation	House wife	381	86.6
	Farmer	15	3.4
	Daily laborer	11	2.5
	Government employee	12	2.7
	Others	21	4.8
Marital status	Married	408	92.7
	Divorced	13	3.0
	Widowed	11	2.5
	Single	8	1.8
Husband's residence	Live together	401	91.1
	Not live together	39	8.9
Husband's educational level	Not educated	59	13.4
	Primary	259	58.9
	Secondary and above	98	22.3
	Unknown	24	5.5
Husband's occupation	Farmer	330	75.0
	Daily laborer	46	10.5
	Government employee	12	2.7
	Others	52	11.8
Total family monthly income	<1000	253	57.5
	≥1000	187	42.5

**Table 2:** Sociodemographic and health service characteristics of under five children in Bereh District, Oromia, Ethiopia, 2021.

Variable	Category	Frequency (*n* = 440)	Percentage (%)
Age of children	≤6 months	22	5.0
	7-11 months	51	11.6
	12-23 months	92	20.9
	24-35 months	172	39.1
	36-47 months	54	12.3
	≥48 months	49	11.1
Sex of children	Male	219	49.8
	Female	221	50.2
Birth order	First child	95	21.6
	The second and above	345	78.4
Children immunized for Rota virus	No	21	4.8
	Yes	419	95.2

**Table 3:** Prevalence of diarrhea among children under five years in Bereh District, Oromia, Ethiopia, 2021.

Variable	Category	Frequency (*n* = 440)	Percentage (%)
Childhood diarrhea	No	364	82.7
	Yes	76	17.3
	Total	440	100.0

**Table 4:** Variables used to check feeding practice among children under five years in Bereh District, Oromia, Ethiopia, 2021.

Variable	Category	Frequency (*n* = 440)	Percentage (%)
Breastfeeding	No	6	1.4
	Yes	434	98.6
Breastfeeding history in the first 6 months	Exclusive	209	47.5
	Not exclusive	231	52.5
Breastfeeding initiation time	Early	212	48.2
	Delayed	228	51.8
Prelacteal feeding	No	399	90.7
	Yes	41	9.3
Duration of EBF	<6 months	237	53.9
	≥6 months	203	46.1
Handwashing at a critical time	No	132	30.0
	Yes	308	70.0
Handwashing method	With water and soap	305	69.3
	Only with water	135	30.7
Source of drinking water	Unimproved	170	38.6
	Improved	270	61.4

**Table 5:** Breastfeeding practice among children under five years in Bereh District, Oromia, Ethiopia, 2021.

Variable	Category	Frequency (*n* = 440)	Percentage (%)
Breastfeeding	No	6	1.4
	Yes	434	98.6
Breastfeeding history in the first 6 months	Exclusive	209	47.5
	Not exclusive	231	52.5
Breastfeeding initiation time	Early	212	48.2
	Delayed	228	51.8
Prelacteal feeding	No	399	90.7
	Yes	41	9.3
Prelacteal feeding ingredients	Nothing	399	90.7
	Butter	12	2.7
	Honey	3	0.7
	Cow's milk	20	4.5
	Others	6	1.4
Duration of EBF	<6 months	237	53.9
	≥6 months	203	46.1
Period of BF continuation	<2 years	254	57.7
	≥2 years	186	42.3

**Table 6:** Complementary feeding practice among children under five years in Bereh District, Oromia, Ethiopia, 2021.

Variable	Category	Frequency (*n* = 440)	Percentage (%)
Age at complementary feeding	<6 months	222	50.5
	At 6 months	152	34.5
	>6 months	50	11.4
	Not started	16	3.6
Prepare child food separately	No	109	24.8
	Yes	331	75.2
Foods children mostly receive	Breast milk	16	3.6
	Cow's milk	70	15.9
	Gruel/soup	70	15.9
	Porridge	182	41.4
	Adults food	87	19.8
	Others	15	3.4
Feeding method	Cup/spoon	219	49.8
	Bottle	87	19.8
	Hand	118	26.8
	Breast	16	3.6

**Table 7:** Hygienic related child feeding practice among children under five years in Bereh District, Oromia, Ethiopia, 2021.

Variable	Category	Frequency (*n* = 440)	Percentage (%)
Feeding unwashed fruits	No	372	84.5
	Yes	68	15.5
Feeding uncooked foods	No	368	83.6
	Yes	72	16.4
Feeding cooked food immediately	No	99	22.5
	Yes	341	77.5
Wash feeding utensils twice or more per a day	No	120	27.3
	Yes	320	72.7
Feeding utensils washing method	Only with water	120	27.3
	With water and soap	320	72.7
Handwashing at a critical time	No	132	30.0
	Yes	308	70.0
Handwashing method	With water and soap	305	69.3
	Only with water	135	30.7
Source of drinking water	Unimproved	170	38.6
	Improved	270	61.4
Method of drinking water treatment at home	Not at all	379	86.1
	Filtering through clothe	30	6.8
	Boiling	22	5.0
	Chemical	3	0.7
	Others	6	1.4

Note: others = water filtering machine.

**Table 8:** Multiple covariate analysis of determinants of under-five diarrhea in Bereh District, Oromia, Ethiopia, 2021.

Variables	Category	COR (95%C.I)	AOR (95%C.I)
Number of under-five children in the house	One	1	1
	Two and above	2.388 (1.376, 4.143)^∗^	2.204 (1.082, 4.488)^∗^
Age of children	≤6 months	0.809 (0.193, 3.395)	2.116 (0.176, 25.449)
	7-11 months	3.043 (1.181, 7.842)^∗^	9.146 (2.055, 40.707)^∗^
	12-23 months	1.334 (0.537, 3.315)	4.979 (1.206, 20.56)^∗^
	24-35 months	0.454 (0.178, 1.156)	1.541 (0.492, 4.833)
	36-47 months	1.625 (0.609, 4.336)	2.630 (0.745, 9.288)
	≥48 months	1	1
Birth order	First child	1	1
	Second and above	0.486 (0.282, 0.838)^∗^	0.137 (0.057, 0.329)^∗∗^
Children immunized for Rota virus	No	1	1
	Yes	0.394 (0.154, 1.013)	1.245 (0.299, 5.183)
Total family size	≤5	1	1
	>5	3.167 (1.878, 5.340)^∗∗^	5.042 (2.326,10.931)^∗∗^
Mother's educational level	Not educated	1	1
	Primary	0.544 (0.310,0.953)^∗^	0.952 (0.453, 2.002)
	Secondary and above	0.314 (0.150,0.657)^∗^	0.486 (0.174, 1.357)
Breastfeeding	No	10.056 (1.808,55.938)^∗^	4.513 (0.489, 41.672)
	Yes	1	1
Breastfeeding history in the first 6 months	Exclusive	1	1
	Not exclusive	2.408 (1.416,4.094)^∗^	4.723 (1.166, 19.134)^∗^
BF initiation time	Early	1	1
	Delayed	2.010 (1.199, 3.368)^∗^	1.382 (0.677, 2.821)
Duration of EBF	<6 months	1.952 (1.160, 3.286)^∗^	0.174 (0.005, 6.668)
	≥6 months	1	1
Period of BF continuation	<2 years	1	1
	≥2 years	0.460 (0.267, 0.793)^∗^	1.456 (0.603, 3.516)
Age at CF	<6 months	1	1
	At 6 months	0.479 (0.270, 0.850)^∗^	0.208 (0.007, 6.402)
	>6 months	0.373 (0.140, 0.988)^∗^	0.242 (0.007, 8.797)
	Not started	0.224 (0.029, 1.733)	0.258 (0.009,7.154)
Prepare child food separately	No	1	1
	Yes	0.425 (0.252,0.717)^∗^	0.252 (0.091, 0.701)^∗^
Feeding method	Cup/spoon	1	1
	Bottle	4.241 (2.215,8.121)^∗∗^	1.655 (0.670, 4.092)
	Hand	3.242 (1.740,6.040)^∗∗^	1.427 (0.486, 4.189)
	Breast	0.663 (0.083,5.287)	
Feeding uncooked foods	No	1	1
	Yes	1.782 (0.975, 3.25)	1.086 (0.486, 2.425)
Feeding cooked food immediately	No	1	1
	Yes	0.310 (0.183,0.525)^∗∗^	0.361 (0.171, 0.762)^∗^
Handwashing at a critical time	No	1	1
	Yes	0.455 (0.274, 0.756)^∗^	0.792 (0.362, 1.729)
Handwashing method	With water and soap	1	1
	Only with water	3.365 (2.024,5.595)^∗∗^	2.949 (1.341, 6.482)^∗^
Source of drinking water	Unimproved	1	1
	Improved	0.360 (0.217,0.598)^∗∗^	0.472 (0.235, 0.949)^∗^
Feeding practice	Poor	1	1
	Good	0.323 (0.185,0.56)^∗∗^	2.694(0.874, 8.303)

Note: 1 = references, ^∗^significant at *p* < 0.05, and ^∗∗^significant at *p* < 0.001.

## Data Availability

The data used to support the findings of this study are included within the supplementary information file.

## Abbreviations

AOR: Adjusted odds ratio
COVID-19: Coronavirus disease
DHS: Demographic and health survey
EBF: Exclusive breastfeeding
EIBF: Early initiation of breastfeeding
EDHS: Ethiopian Demographic and Health Survey
HSTP: Health Sector Transformation Program
IYCF: Infant and young child feeding
LMIC: Low- and middle-income country
MCH: Maternal and child health
NGO: Nongovernmental organization
OR: Odds ratio
SDG: Sustainable Development Goal
SPSS: Statistical Package for the Social Science
UNICEF: United Nation International Children's Emergency Fund
WHO: World Health Organization.

## Supplementary Materials

Supplementary Materials Bivariate analysis of sociodemographic and health service determinants of under-five diarrhea in Bereh woreda, Oromia, Ethiopia, 2021. Bivariate analysis of child feeding practice determinant of under-five diarrhea in Bereh woreda, Oromia, Ethiopia, 2021. Bivariate analysis of breastfeeding practice determinants of under-five diarrhea in Bereh woreda, Oromia, Ethiopia, 2021. Bivariate analysis of complementary feeding practice determinants of under-five diarrhea in Bereh woreda, Oromia, Ethiopia, 2021. Bivariate analysis of hygienic related child feeding practice determinants of under-five diarrhea in Bereh woreda, Oromia, Ethiopia, 2021.
