# Supplementary material for: Prevalence of Diarrhea, Feeding Practice, and Associated Factors among Children under Five Years in Bereh District, Oromia, Ethiopia
Source: Infect Dis Obstet Gynecol. 2022 Jun 17;2022:4139648. doi: 10.1155/2022/4139648 (PMC9232332; doi:10.1155/2022/4139648)
Supplement: Supplementary Materials — Bivariate analysis of sociodemographic and health service determinants of under-five diarrhea in Bereh woreda, Oromia, Ethiopia, 2021. Bivariate analysis of child feeding practice determinant of under-five diarrhea in Bereh woreda, Oromia, Ethiopia, 2021. Bivariate analysis of breastfeeding practice determinants of under-five diarrhea in Bereh woreda, Oromia, Ethiopia, 2021. Bivariate analysis of complementary feeding practice determinants of under-five diarrhea in Bereh woreda, Oromia, Ethiopia, 2021. Bivariate analysis of hygienic related child feeding practice determinants of under-five diarrhea in Bereh woreda, Oromia, Ethiopia, 2021. [file 4139648.f1.docx]

Bivariate analysis of socio-demographic and health service determinants of under-five diarrhea in Bereh woreda, Oromia, Ethiopia, 2021

| Variable | **Category** | **Diarrhea** | | **COR(95%C.I)** |
| --- | --- | --- | --- | --- |
|  |  | **No n(%)** | **Yes n(%)** |  |
| Number of under five children in the house | One | 302(68.6) | 51(11.6) | 1 |
|  | Two and above | 62(14.1) | 25(5.7) | 2.388(1.376,4.143)* |
| Age of children | ≤ 6 months | 19(4.3) | 3(0.7) | 0.809(0.193,3.395) |
|  | 7-11 months | 32(7.3) | 19(4.3) | 3.043(1.181,7.842)* |
|  | 12-23 months | 73(16.6) | 19(4.3) | 1.334(0.537,3.315) |
|  | 24-35 months | 158(35.9) | 14(3.2) | 0.454(0.178,1.156) |
|  | 36-47 months | 41(9.3) | 13(3.0) | 1.625(0.609,4.336) |
|  | ≥ 48 months | 41(9.3) | 8(1.8) | 1 |
| Sex of children | Male | 180(40.9) | 39(8.9) | 1 |
|  | Female | 184(41.8) | 37(8.4) | 0.928(0.566, 1.522) |
| Birth order | First child | 70(15.9) | 25(5.7 | 1 |
|  | second and above | 294(66.8) | 51(11.6) | 0.486(0.282,0.838)* |
| Children immunized for Rota virus | No | 14(3.2) | 7(1.6) | 1 |
|  | Yes | 350(79.5) | 69(15.7) | 0.394(0.154, 1.013)*** |
| ANC follow up | No | 21(4.8) | 6(1.4) | 1 |
|  | Yes | 343(78.0) | 70(15.9) | 0.714(0.278, 1.834) |
| Place of delivery | Home | 121(27.5) | 26(5.9) | 1 |
|  | Health institution | 234(53.2) | 49(11.1) | 0.975(0.577, 1.645) |
|  | Others | 9(2.0) | 1(0.2) | 0.517(0.063, 4.261) |
| Mothers/care givers age | ≤ 24 years | 60(13.6) | 13(3.0) | 1 |
|  | 25-34 years | 233(53.0) | 47(10.7) | 0.931(0.473, 1.831) |
|  | ≥ 35 years | 71(16.1) | 16(3.6) | 1.040(0.463, 2.335) |
| Ethnicity | Oromo | 316(71.8) | 68(15.5) | 1 |
|  | Amhara | 30(6.8) | 6(1.4) | 0.929(0.372, 2.320) |
|  | Others | 18(4.1) | 2(0.5) | 0.516(0.117, 2.278) |
| Religion | Orthodox | 310(70.5) | 65(14.8) | 1 |
|  | Muslim | 12(2.7) | 4(0.9) | 1.590(0.497, 5.085) |
|  | Protestant | 7(1.6) | 2(0.5) | 1.363(0.277, 6.709) |
|  | Others | 35(8.0) | 5(1.1) | 0.681(0.257, 1.805) |
| Mother/care givers educational level | Not educated | 77(17.5) | 28(6.4) | 1 |
|  | Primary | 182(41.4) | 36(8.2) | 0.544(0.310,0.953)* |
|  | Secondary and above | 105(23.9) | 12(2.7) | 0.314(0.150,0.657)* |
| Mother's occupation | House wife | 318(72.3) | 63(14.3) | 1 |
|  | Farmer | 11(2.5) | 4(0.9) | 1.835(0.566, 5.949) |
|  | Daily laborer | 9(2.0) | 2(0.5) | 1.122(0.237, 5.316) |
|  | Government employee | 9(2.0) | 3(0.7) | 1.683(0.443, 6.389) |
|  | Others | 17(3.9) | 4(0.9) | 1.188(0.387, 3.648) |
| Marital status | Married | 339(77.0) | 69(15.7) | 1 |
|  | Divorced | 10(2.3) | 3(0.7) | 1.474(0.395, 5.495) |
|  | Widowed | 9(2.0) | 2(0.5) | 1.092(0.231, 5.164) |
|  | Single | 6(1.4) | 2(0.5) | 1.638(0.324, 8.284) |
| Total family size | ≤ 5 | 293(66.6) | 43(9.8) | 1 |
|  | > 5 | 71(16.1) | 33(7.5) | 3.167(1.878,5.340)** |
| Husband's residence | Live together | 334(75.9) | 67(15.2) | 1 |
|  | Not live together | 30(6.9) | 9(2.0) | 1.496(0.679, 3.294) |
| Husband's educational level | Not educated | 48(10.9) | 11(2.5) | 1 |
|  | Primary | 218(49.5) | 41(9.3) | 0.821(0.393, 1.712) |
|  | Secondary and above | 79(18.0) | 19(4.3) | 1.049(0.460, 2.394) |
|  | Unknown | 19(4.3) | 5(1.1) | 1.148(0.352, 3.749) |
| Husband's occupation | Farmer | 273(62.0) | 57(13.0) | 1 |
|  | Daily laborer | 37(8.4) | 9(2.0) | 1.165(0.533, 2.547) |
|  | Government employee | 9(2.0) | 3(0.7) | 1.596(0.419, 6.082) |
|  | Others | 45(10.2) | 7(1.6) | 0.745(0.320, 1.736) |
| Total family monthly income | < 1000 | 206(46.8) | 47(10.7) | 1 |
|  | ≥ 1000 | 158(35.9) | 29(6.6) | 0.804(0.484, 1.336) |

Note 1=references, *significant at p <0.05, **significant at p <0.001, ***significant at p <0.25

Bivariate analysis of child feeding practice determinant of under-five diarrhea in Bereh woreda, Oromia, Ethiopia, 2021

| Variable | **Category** | **Diarrhea** | | **COR(95%C.I)** |
| --- | --- | --- | --- | --- |
|  |  | **No n(%)** | **Yes n(%)** |  |
| Feeding practice | Poor | 179(40.7) | 57(13.0) | 1 |
|  | Good | 185(42.0) | 19(4.3) | 0.323(0.185,0.56)^**^ |

Note 1=references, *significant at p <0.05, **significant at p <0.001

Bivariate analysis of breast feeding practice determinants of under-five diarrhea in Bereh woreda, Oromia, Ethiopia, 2021

| Variable | | **Category** | **Diarrhea** | | **COR(95%C.I)** |
| --- | --- | --- | --- | --- | --- |
|  |  |  | **No n(%)** | **Yes n(%)** |  |
| Breast feeding | | No | 2(0.5) | 4(0.9) | 10.056(1.808, 55.938)* |
|  | | Yes | 362(82.3) | 72((16.4) | 1 |
| Breastfeeding history in the first 6 months | Exclusive | | 186(42.3) | 23(5.2) | 1 |
|  | Not exclusive | | 178(40.5) | 53(12.0) | 2.408(1.416, 4.094)* |
| Breast feeding initiation time | Early | | 186(42.3) | 26(5.9) | 1 |
|  | Delayed | | 178(40.5) | 50(11.4) | 2.010(1.199, 3.368)* |
| Prelacteal feeding | No | | 332(75.5) | 67(15.2) | 1 |
|  | Yes | | 32(7.3) | 9(2.0) | 1.394(0.636, 3.055) |
| Prelacteal feeding ingredients | Nothing | | 332(75.5) | 67(15.2) | 1 |
|  | Butter | | 9(2.0) | 3(0.7) | 1.652(0.436, 6.262) |
|  | Honey | | 2(0.5) | 1(0.2) | 2.478(0.221, 27.717) |
|  | Cow's milk | | 16(3.6) | 4(0.9) | 1.239(0.402, 3.822) |
|  | Others | | 5(1.1) | 1(0.1) | 0.991(0.114, 8.619) |
| Duration of EBF | < 6 months | | 186(42.3) | 51(11.6) | 1.952(1.160, 3.286)* |
|  | ≥ 6 months | | 178(40.5) | 25(5.7) | 1 |
| Period of BF continuation | < 2 years | | 199(45.2) | 55(12.5) | 1 |
|  | ≥ 2 years | | 165(37.5) | 21(4.5) | 0.460(0.267, 0.793)* |

Note 1=references, *significant at p <0.05, **significant at p <0.001

Bivariate analysis of complementary feeding practice determinants of under-five diarrhea in Bereh woreda, Oromia, Ethiopia, 2021

| Variable | | **Category** | **Diarrhea** | | **COR(95%C.I)** |
| --- | --- | --- | --- | --- | --- |
|  |  |  | **No n(%)** | **Yes n(%)** |  |
| Age at complementary feeding | < 6 months | | 171(38.9) | 51(11.6) | 1 |
|  | At 6 months | | 133(30.2) | 19(4.3) | 0.479(0.270, 0.850)* |
|  | > 6 months | | 45(10.2) | 5(1.1) | 0.373(0.140, 0.988)* |
|  | Not started | | 15(3.4) | 1(0.2) | 0.224(0.029, 1.733) |
| Prepare child food separately | No | | 79(18.0) | 30(6.8) | 1 |
|  | Yes | | 285(64.8) | 46(10.5) | 0.425(0.252,0.717)* |
| Foods child mostly receive | Breast milk | | 15(3.4) | 1(0.2) | 1 |
|  | Cow's milk | | 57(13.0) | 13(3.0) | 3.421(0.414, 28.274) |
|  | Gruel/soup | | 59(13.4) | 11(2.5) | 2.797(0.334, 23.395) |
|  | Porridge | | 150(34.1) | 32(7.3) | 3.200(0.408, 25.105) |
|  | Adults food | | 71(16.1) | 16(3.6) | 3.380(0.416, 27.484) |
|  | Others | | 12(2.7) | 3(0.7) | 3.750(0.345, 40.806) |
| Feeding method | Cup/spoon | | 199(45.2) | 20(4.5) | 1 |
|  | Bottle | | 61(13.9) | 26(5.9) | 4.241(2.215,8.121)** |
|  | Hand | | 89(20.2) | 29(6.6) | 3.242(1.740,6.040)** |
|  | Breast | | 15(3.4) | 1(0.2) | 0.663(0.083,5.287) |

Note 1=references, *significant at p <0.05, **significant at p <0.001

Bivariate analysis of hygienic related child feeding practice determinants of under-five diarrhea in Bereh woreda, Oromia, Ethiopia, 2021

| Variable | | **Category** | **Diarrhea** | | **COR(95%C.I)** |
| --- | --- | --- | --- | --- | --- |
|  |  |  | **No n(%)** | **Yes n(%)** |  |
| Feeding unwashed fruits | No | | 310(70.5) | 62(14.1) | 1 |
|  | Yes | | 54(12.3) | 14(3.2) | 1.296(0.678, 2.478) |
| Feeding uncooked foods | No | | 310(70.5) | 58(13.2) | 1 |
|  | Yes | | 54(12.3) | 18(4.1) | 1.782(0.975, 3.25)*** |
| Feeding cooked foods immediately | No | | 67(15.2) | 32(7.3) | 1 |
|  | Yes | | 297(67.5) | 44(10.0) | 0.310(0.183, 0.525)** |
| wash feeding utensils twice or more per a day | No | | 97(22.0) | 23(5.2) | 1 |
|  | Yes | | 267(60.7) | 53(12.0) | 0.837(0.487, 1.439) |
| Feeding utensils washing method | Only with water | | 97(22.0) | 23(5.2) | 1 |
|  | With water and soap | | 267(60.7) | 53(12.0) | 0.837(0.487, 1.439) |
| Hand washing at critical time | No | | 98(22.3) | 34(7.7) | 1 |
|  | Yes | | 266(60.5) | 42(9.5) | 0.455(0.274, 0.756)* |
| Hand washing method | With water and soap | | 270(61.4) | 35(8.0) | 1 |
|  | Only with water | | 94(21.4) | 41(9.3) | 3.365(2.024, 5.595)** |
| Source of drinking | Unimproved | | 125(28.4) | 45(10.2) | 1 |
| water | Improved | | 239(54.3) | 31(7.0) | 0.360(0.217, 0.598)** |
| Method of drinking water treatment at home | Not at all | | 315(71.6) | 64(14.5) | 1 |
|  | Filtering through clothe | | 24(5.5) | 6(1.4) | 1.230(0.484, 3.131) |
|  | Boiling | | 18(4.1) | 4(0.9) | 1.094(0.358, 3.340) |
|  | Chemical | | 2(0.5) | 1(0.2) | 2.461(0.220, 27.550) |
|  | Others | | 5(1.1) | 1(0.2) | 0.984(0.113, 8.568) |

Note 1=references, *significant at p <0.05, **significant at p <0.001, ***significant at p <0.25
